# Robust Resource Control Based on AP Selection in 6G-Enabled IoT Networks

**DOI:** 10.3390/s23156788

**Published:** 2023-07-29

**Authors:** Ashu Taneja, Ali Alqahtani, Nitin Saluja, Nayef Alqahtani

**Affiliations:** 1 Chitkara University Institute of Engineering and Technology, Chitkara University, Rajpura 140401, Punjab, India; nitin.saluja@chitkara.edu.in; 2 Department of Networks and Communications Engineering, College of Computer Science and Information Systems, Najran University, Najran 61441, Najran, Saudi Arabia; asalqahtany@nu.edu.sa; 3 Department of Electrical Engineering, College of Engineering, King Faisal University, Al-Hofuf 31982, Al-Ahsa, Saudi Arabia; nmalqahtani@kfu.edu.sa

**Keywords:** 6G, cell-free framework, PBAS algorithm, spectral efficiency, receive combining

## Abstract

The diverse application vertices of internet-of-things (IoT) including internet of vehicles (IoV), industrial IoT (IIoT) and internet of drones things (IoDT) involve intelligent communication between the massive number of objects around us. This digital transformation strives for seamless data flow, uninterrupted communication capabilities, low latency and ultra-high reliability. The limited capabilities of fifth generation (5G) technology have given way to sixth generation (6G) wireless technology. This paper presents a dynamic cell-free framework for a 6G-enabled IoT network. A number of access points (APs) are distributed over a given geographical area to serve a large number of user nodes. A pilot-based AP selection (PBAS) algorithm is proposed, which offers robust resource control through AP selection based on pilots. Selecting a subset of APs against all APs for each user node results in improved performance. In this paper, the performance of the proposed transmission model is evaluated for the achieved data rate and spectral efficiency using the proposed algorithm. It is shown that the proposed PBAS algorithm improves the spectral efficiency by 22% at the cell-edge and 1.5% at the cell-center. A comparison of the different combining techniques used at different user locations is also provided, along with the mathematical formulations. Finally, the proposed model is compared with two other transmission models for performance evaluation. It is observed that the spectral efficiency achieved by an edge node with the proposed scheme is 5.3676 bits/s/Hz, compared to 0.756 bits/s/Hz and 1.0501 bits/s/Hz, attained with transmission schemes 1 and 2, respectively.

## 1. Introduction

### 1.1. IoT and 6G: Background and Motivation

The revolutionary increase in the number of connected devices, networked connections, network services and mobile data traffic has placed a huge burden on network service providers. With this internet-of-things (IoT) revolution, it is becoming very challenging to meet the communication needs of the expanding IoT ecosystem. The IoT is the basis of the digital transformation of people, things, organizations, cities and industries, with application in diverse verticles. The communication support between the vehicles in V2V communication, as well as that between the vehicular nodes and the infrastructure in V2I communication, is very crucial for the internet-of-vehicles (IoV) [1]. Similarly, the internet-of-medical things (IoMT) [2], internet-of-underwater things (IoUT) [3], and internet-of-drone things (IoDT) [4] are some of the other IoT application domains that depend on extended communication capabilities with low latency and ultra-high reliability [5,6]. The limited ability of the fifth generation (5G) to meet the requirements of these diverse applications led to the development of new technologies, along with the evolution of current technologies. This has given rise to a new dimension, beyond 5G and sixth generation (6G) wireless technology. 6G aims to provide faster data speeds than 5G, offering terabits-per-second (Tbps) peak data rates. It can provide extended network coverage, even to remote areas, under harsh propagation environments or at cell edges. These future wireless technologies aim to achieve a network intelligentization that is capable of handling extreme data traffic, event-defined service capabilities with low latency and ultra-high reliability, as well as computation-intensive communication with targeted computational accuracy [7].

### 1.2. Emerging 6G Technologies and Their Limitations

The potential breakthrough technologies in the movement from 5G to the 6G era include millimeter wave (mmWave), heterogeneous networks (HetNets) and multiple antenna technologies, including massive multiple-input–multiple-output (MIMO) and cell-free massive MIMO and intelligent reflecting surfaces (IRS) [8]. These multi-antenna technologies can be integrated with IRS technology and unmanned aerial vehicle (UAV) technology to support IRS-UAV-assisted communication [9,10]. Different UAV-to-ground (U2G) communication scenarios, including UAV-to-vehicle (U2V), are supported by 6G to offer reliable communication [11,12]. MmWave communication offers large unused bandwidths but the drawbacks of shorter wavelengths, signal degradation, reduced coherence time and signaling overhead require more research efforts for its use in next-generation wireless systems [13,14]. The densification of cells in HetNets improves the data rate [15], but the small cell density should not exceed a critical threshold. To reap all the benefits of small cells, the optimal leveraging of small cells is required [16]. Massive MIMO is a technology in which each base station (BS) is equipped with a large number of antennas (100 or more) that serve all the users in a given geographical area [17,18]. Although it offers beamforming and spatial multiplexing gains, the main drawback is cell edge traffic congestion. Due to the insufficient data rates achieved at the cell edge, the user experience defined by user data rates guaranteed to 95% of the users is mediocre in the massive MIMO networks. Moreover, a high transmit power and increased energy overhead are the other challenges. The inter-cell interference and subsequent call drops due to handovers limit its use for heavy-traffic networks [19]. As an alternative, cell-free massive MIMO has gained a lot of attention, in which a large number of access points (APs) are distributed, which serve large number of users [20]. Each user is assigned a subset of APs that cooperates with each other in order to connect with a user. In this user-centric approach, the user decides which set of APs best connects with minimum interference [21,22]. This is in contrast to the network-centric approach where the network is divided into clusters, with a few APs in each cluster [23]. However, the handover and interference issues at the cluster edges remain unaddressed, which are actively taken care of in the cell-free user-centric approach.

### 1.3. Related Work

In the 6G-enabled IoT networks, massive MIMO technology has already been applied to support a larger number of IoT devices, sensors and other connected nodes. The incorporation of massive MIMO in the IIoT applications is explored in [24,25,26,27] with a powerful BS at the data center to maintain information flow between different user entities. The more transmitters there are, the higher the number of devices it can support. However, the high complexity and implementation overhead in real-time environments are the challenges that limit its use. The cell-free approach was recently introduced to provide massive connectivity to end users in an IoT network. A large number of APs are geographically distributed to serve a large number of users, which are connected to cloud-edge processors called central processing units (CPUs) [28]. The information from the APs is gathered by the CPUs, which are utilized for encoding and decoding. The performance of a cell-free massive MIMO is evaluated for spectral efficiency in [29], where each user and AP are equipped with a single antenna. The spectral efficiency achieved by 95% of the users increased to five times of that using the cell-free approach. This can be increased further by equipping each user with multiple antennas and AP [30,31,32].

The cell-free massive MIMO communication model is considered for energy self-sustainability in 6G internet-of-everything (IoE) networks [33]. It utilizes the concept of energy harvesting and power control to overcome the total energy overhead. Ref. [34] considers cell-free massive MIMO for simultaneous wireless information and power transfer (SWIPT) in IoT networks. Conjugate beamforming is used, such that the APs direct the radio frequency (RF) energy toward the functional IoT nodes. Ref. [35] proposes a modified conjugate beamforming such that the self-interference is eliminated and the performance is enhanced. Refs. [36,37] suggest that the energy efficiency can be improved by ten times in a cell-free massive MIMO system as compared to the cellular massive MIMO system. The cell-free massive MIMO system with geographically separated antennas is compared with the conventional massive MIMO system model, which uses collocated antennas in [38]. The cell-free approach has a superior performance and is more suitable for providing massive connectivity. The performance of the cell-free massive MIMO system subject to hardware constraints is evaluated in [39] with spectral efficiency as the primary parameter. The effects of hardware imperfections owing to the large number of distributed transceivers are discussed in [40].

The literature contains a number of papers [41,42,43,44] that deal with signal processing in cell-free massive MIMO networks in different fading environments. A system performance evaluation for Rayleigh fading is carried out in [45], for Rician fading channel in [46,47], while channel correlation is considered in [48]. The different receivers are compared in [41], where it is shown that, in a cell-free network, minimum mean square error (MMSE) and large-scale fading decoding (LSFD) receivers outperform the matched filter receiver, achieving a gain performance of five-fold and two-fold, respectively. The pilot assignment, transmit precoding and receive combining are highlighted in [42,43], which exploits the cooperative dynamic clustering used in network MIMO. The performance of partial zero-forcing precoding is evaluated in [44] for a cell-free network. Ref. [49] uses clustering-based algorithms to improve the activity detection performance of a cell-free massive MIMO network. Ref. [50] exploits the channel knowledge and proposes a power control algorithm such that the computational time is reduced. This results in a scalable and energy-efficient IoT network. Ref. [51] provides a solution to the constraint of limited fronthaul bandwidth in the uplink of cell-free massive MIMO systems by proposing a novel framework of the joint optimization of AP scheduling and power control. Ref. [52] proposes two receiver designs to overcome the constraint of rate-limited fronthaul.

In this paper, a dynamic cell-free framework is proposed for a 6G-enabled IoT network, which aims for improved network connectivity and network coverage. A communication scenario is considered where a large number of APs are located in a geographical area to serve the massive communicating nodes. Each node connects with a subset of APs, which are selected by the proposed algorithm, namely PBAS, the pilot-based AP selection algorithm. The system model’s performance is evaluated in terms of the achieved data rates and spectral efficiency.

### 1.4. Contributions and Outcomes

The IoT ecosystem is expanding with intelligent communication nodes, which need to seamlessly exchange information. Providing reliable communication support to these interconnected nodes is the main challenge in future IoT networks. This paper offers a robust network solution powered by 6G such that all the nodes, including the edge nodes, are provided with uniform network coverage. The novel contributions of the paper are:

- A dynamic cell-free framework is presented for the 6G-powered communication model, where a large number of APs cooperate to serve the IoT nodes. In conventional systems, all APs serve all the nodes in the network, while in the proposed scenario, a set of APs serve a user node.
- To enable efficient resource utilization, an AP selection algorithm, PBAS, is proposed, which allocates a set of APs to each node. The selection of APs is based on the allocation of pilots to the nodes with minimum pilot interference at the AP.
- A spectral efficiency analysis is carried out with detailed mathematical formulations of the signal processing that is involved, which includes channel estimation and receive-combining. The system performance is evaluated for the achieved spectral efficiency for different user transmit powers at varied locations.
- The performance of optimal and scalable receive combiners is also discussed in terms of the achieved spectral efficiency.
- To validate the performance evaluation, the proposed system is also compared with the other two system models, one in which all APs serve a user and the other in which one AP serves one user.

## 2. System Model

**Figure 1:**
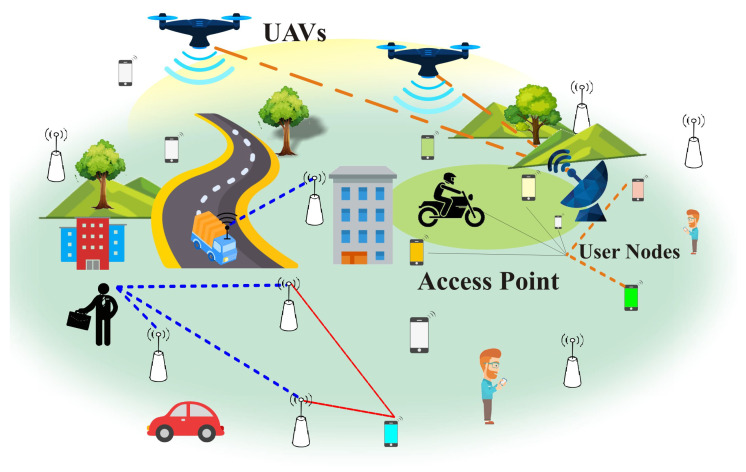
Proposed communication model, where *N* number of APs are distributed to serve a number of *K* users.

This section presents the communication model, which is based on a cell-free network scenario. A total of *K* mobile nodes are distributed in a given geographical area, where *N* access points (APs) are randomly deployed. Figure 1 illustrates the proposed communication scenario. The APs cooperate among themselves to jointly serve the user’s nodes in a coverage area. Each AP is equipped with *M* antennas. The APs are connected to the CPU via fronthaul links. Time division duplex (TDD) mode is used for the network operation, where uplink pilots are used to acquire channel state information (CSI) between AP *n* and node *k*. The user nodes send pilot signals in the uplink training phase, which enable the AP *n* to provide the channel estimates to all users. Let $h_{kn}$ be the channel coefficients between AP *n* and user node *k*, which follows correlated Rayleigh fading distribution such that $h_{kn}∼N\left(0_{M},R_{kn}\right)$, $R_{kn}$ is the spatial correlation matrix, which is related to the average channel gains $\delta_{kn}$ as

(1) $$\delta_{kn}=\frac{1}{N}tr\left(R_{kn}\right)$$

Additionally, $R_{kn}=E\left\{h_{kn}h_{kn}^{H}\right\}$.

A high-user-mobility urban environment is considered with large-scale fading computed according to the 3GPP model, as defined in [53].

(2) $$\delta_{kn}\left[dB\right]=-30.5-36.7log_{10}\left(\frac{r_{kn}}{1m}\right)+S_{kn}$$

This is dependent on the distance between the user nodes and the APs $r_{kn}$ and shadow fading $S_{kn}$. It is assumed that the wireless APs are sufficiently separated such that the channel elements to the different APs are independent $E\left\{h_{kl}h_{kn}^{H}\right\}=0,n\neq l$.

The system considers block fading model in the uplink where the training symbols and payload data are transmitted in the duration $t_{ul}$ of coherence block of interval $t_{c}$ such that $t_{c}=\left(t_{ul}+t_{dl}\right)$, $t_{dl}$, with this being the downlink transmission duration, $t_{ul}=\left(t_{p}+t_{d}\right)$ being the sum of training symbol duration $t_{p}$, and data duration being $t_{d}$. The coherence block contains $\tau_{c}$ symbols with coherence bandwidth $B_{c}$.

### 2.1. Pilot Transmission and Channel Estimation

Let $\psi =\left\{\psi_{1},\psi_{2}\ldots \psi_{t_{p}}\right\}$ be a set of $t_{p}$ pilot sequences that are mutually orthogonal such that the interference between the user transmissions is negligible. The pilot assigned to user node *k* has the index $t_{k}\in \left\{1,2,\ldots t_{p}\right\}$ and $U_{k}$ defines a set of user nodes that are assigned the same pilot as that of node *k*. The pilot signal received at AP *n* is given as follows:

(3) $$y_{n}^{p}=\;\;\sum_{k=1}^{K}\sqrt{\alpha_{k}}\;h_{kn\;}\psi_{t_{k}\;}^{T}+\;N_{n}$$

where $N_{n}$ is the receiver noise, and $\alpha_{k}$ is the pilot transmit power of user node *k*. These pilots are used to estimate the channel coefficients ${\hat{h}}_{kn}:k\;=\;1,\;\ldots .,K,\;n\;\epsilon \;S_{k}$

MMSE channel estimation is used to evaluate ${\hat{h}}_{kn}$

(4) $${\hat{h}}_{kn}=\;\sqrt{\alpha_{k}t_{p}}R_{kn}\varphi_{t_{k}n}^{-1}\;{\bar{y}}_{t_{k}n}^{p}\;$$

where ${\bar{y}}_{t_{k}n}^{p}=y_{n}^{p}\psi_{t_{k}n}^{*}/{\sqrt{t}}_{p}$ and $R_{kn}$ is the spatial correlation matrix, which is equal to $E\left\{h_{kn}h_{kn}^{H}\right\}$, $\varphi_{t_{k}n}=E\left\{{\bar{y}}_{t_{k}n}^{p}{\left({\bar{y}}_{t_{k}n}^{p}\right)}^{H}\right\}=$$\sum_{i\varepsilon U_{k}}$ $\alpha_{i}t_{p}R_{in}+\sigma^{2}I_{M}$ is the correlation matrix of the received signal, and $C_{kn}=E\left\{{\tilde{h}}_{kn\;}{\tilde{h}}_{kn}^{H}\right\}=R_{kn}-\alpha_{k}t_{p}R_{kn}\psi_{t_{k}n}^{-1}R_{kn}$ is the error correlation matrix and ${\tilde{h}}_{kn\;}=h_{kn}-{\hat{h}}_{kn}$.

The estimate ${\hat{h}}_{k}=\;{\left[{\hat{h}}_{k1}^{T},\;{\hat{h}}_{k2}^{T}\ldots \ldots {\hat{h}}_{kN}^{T}\right]}^{T}$ of the collective channel $h_{k}$ can be obtained as follows:

(5) $$\beta_{k}{\hat{h}}_{k}=\left[\begin{array}{c} \beta_{k1}{\hat{h}}_{k1}\\ \beta_{k2}{\hat{h}}_{k2}\\ \begin{array}{c} \vdots \\ \beta_{kN}{\hat{h}}_{kN} \end{array} \end{array}\right]∼N\left(0_{MN},\alpha_{k}t_{p}\beta_{k}R_{k}\;\psi_{t_{k}}^{-1}R_{k}\beta_{k}\right)$$

where $\beta_{k}=diag\left(\beta_{k1},\ldots \beta_{kN}\right)$, such that

(6) $$\beta_{kn}=\left\{\;\;\;\begin{array}{c} I_{M}\;\;\;\;\;\;\;\;n\epsilon S_{k}\\ \;0\;\;\;\;\;n\notin S_{k}\;\;\;\;\;\;\;\; \end{array}\right\}$$

$R_{k}=diag\left(R_{k1},\ldots R_{kN}\right)$ and $\psi_{t_{k}}^{-1}=diag\left(\psi_{t_{k}1}^{-1},\ldots \psi_{t_{k}N}^{-1}\right)$ are the collective correlation matrices.

### 2.2. Data Transmission and Data Detection

During data transmission phase, data are sent by all the nodes whose superposition is received by all the APs. The data signal received by the $n^{th}$ AP is given by

(7) $$y_{n}\;\;=\;\sum_{k=1}^{K}\sqrt{\alpha_{k}}h_{kn}s_{k}\;\;+\;n_{n}$$

where $s_{k}$ is the signal transmitted by node *k* in the uplink, and $n_{n}$ is the receiver noise. It is assumed that the pilot transmit power and data transmit power of each AP is same. The overall received signal in the uplink is

(8) $$Y=\left[\begin{array}{c} y_{1}\\ y_{2}\\ \begin{array}{c} \vdots \\ y_{n} \end{array} \end{array}\right]=\sum_{k=1}^{K}\sqrt{\alpha_{k}}h_{k}s_{k}+n_{n}$$

In the considered system model based on a cell-free approach, two different network operations are evaluated. In the centralized operation, each AP sends the received pilot and data signals to the CPU where the channel is estimated, and data are detected using receive-combining. Another form of operation is the distributed operation in which the channel estimates are obtained locally at the AP to obtain the signal estimates, which are sent to the CPU for final data detection. Let ${\hat{s}}_{k}$ be the obtained signal estimates

(9) $${\hat{s}}_{k}=\sum_{n=1}^{N}{\hat{s}}_{kn}=\sum_{n=1}^{N}c_{kn}^{H}\beta_{kn}y_{n}$$

The signal estimates are computed using a receive-combiner vector $c_{kn}$.

(10) $${\hat{s}}_{k}=\sum_{n=1}^{N}c_{kn}^{H}\beta_{kn}y_{n}$$

Since, in the considered system model, only a subset of APs $S_{k}$ are serving a particular node *k*, only those APs $n\epsilon S_{k}$ will contribute to the node *k* data estimation.

(11) $${\hat{s}}_{kn}=\sum_{n\epsilon S_{k}}c_{kn}^{H}y_{n}$$

For a user node *k*, the achievable spectral efficiency is given by

(12) $${SE}_{k}=\;{log}_{2}\left(1+\;\Upsilon_{k}\right)$$

(13) $$\Upsilon_{k}=\frac{\alpha_{k}\;{\left|c_{k}^{H}\beta_{k}{\hat{h}}_{k}\right|}^{2}}{\sum_{i=1,i\neq k}^{K}\alpha_{i}\;|c_{k}^{H}\beta_{k}{\hat{h}}_{i}{|}^{2}+c_{k}^{H}\left(\sum_{i=1}^{K}\alpha_{i}\beta_{k}C_{i}\beta_{k}\right)c_{k}+\sigma^{2}\left|\right|\beta_{k}c_{k}{\left|\right|}^{2}}$$

with $C_{i}$ being the collective error correlation matrix. The mean square error (MSE) is given by

(14) $$MSE=E\left\{|s_{k}-{\hat{s}}_{k}{|}^{2}\right\}$$

The optimal receive-combining scheme, which maximizes $\Upsilon_{k}$ and minimises MSE is the minimum mean square error (MMSE) receive-combining, is given as

(15) $$c_{k}^{MMSE}=\alpha_{k}{\left(\sum_{i=1}^{K}\alpha_{i}\beta_{k}\left({\hat{h}}_{i}{\hat{h}}_{i}^{H}+C_{i}\right)\beta_{k}+\sigma^{2}I_{NM}\right)}^{-1}\beta_{k}{\hat{h}}_{k}$$

The receive-combining schemes for the centralized network operation utilised for data detection need to be less computationally complex and scalable. The maximal ratio (MR) combining scheme is one such scheme with low complexity, and the complexity is independent of the number of user nodes *K*, which is defined as follows

(16) $$c_{k}^{MR}=\beta_{k}{\hat{h}}_{k}$$

Another scheme is the partial MMSE scheme, which is a subcase of MMSE receive-combining where the APs in set $S_{k}$ serve a node *k* against all the APs.

(17) $$c_{k}^{PMMSE}=\alpha_{k}{\left(\sum_{i\in S_{k}}\alpha_{i}\beta_{k}{\hat{h}}_{i}{\hat{h}}_{i}^{H}\beta_{k}+Z_{S_{k}}+\sigma^{2}I_{NM}\right)}^{-1}\beta_{k}{\hat{h}}_{k}$$

with $Z_{s_{\kappa }}=\sum_{i\in S_{k}}\alpha_{i}\beta_{k}C_{i}\beta_{k}$.

Since the PMMSE-combining scheme depends on $S_{k}$, the computational complexity depends on $|S_{k}|$. To overcome this, partial regularized zero-forcing (PRZF) receive combining is obtained by neglecting the term $Z_{S_{k}}$ in Equation (15).

(18) $$c_{k}^{PRZF}=\alpha_{k}{\left(\sum_{i\in S_{k}}\alpha_{i}\beta_{k}{\hat{h}}_{i}{\hat{h}}_{i}^{H}\beta_{k}+\sigma^{2}I_{NM}\right)}^{-1}\beta_{k}{\hat{h}}_{k}$$

## 3. AP Selection

With the expanding network traffic due to the large number of connected devices, frequent call drops, transmission delays, network congestion and a lack of reliable communication are the main challenges of future wireless networks. 6G-enabled wireless networks aim for seamless network connectivity, low latency and ultra-high reliability. The effective utilization of network resources, which include the number of APs, number of user nodes, number of antennas, and relays, is very important to achieve sustainable future networks [54]. In this paper, the proposed communication model involves a large number of APs being deployed in a given geographical area to serve a large number of user nodes. Conventionally, all APs serve all the user nodes communicating in that area [30]. With the introduction of AP selection, it is ensured that, against all APs, a subset of APs serve a given node. This also results in the efficient management of hardware resources leading to reduced energy overheads [55].

This section introduces the proposed AP selection algorithm, namely pilot-based AP selection (PBAS), for the proposed communication model defined in Section 2. In a given geographical area, a number of *N* APs are distributed to serve the number of *K* users residing in that area. In contrast to conventional cellular systems, in which all APs serve each user node, the proposed cell-free approach uses a subset of APs that cooperate among themselves to serve a user node *k*. The proposed algorithm defines a subset of APs $S_{k}$ for communication with every user node *k*, as given below.

### PBAS Algorithm

**Figure 2:**
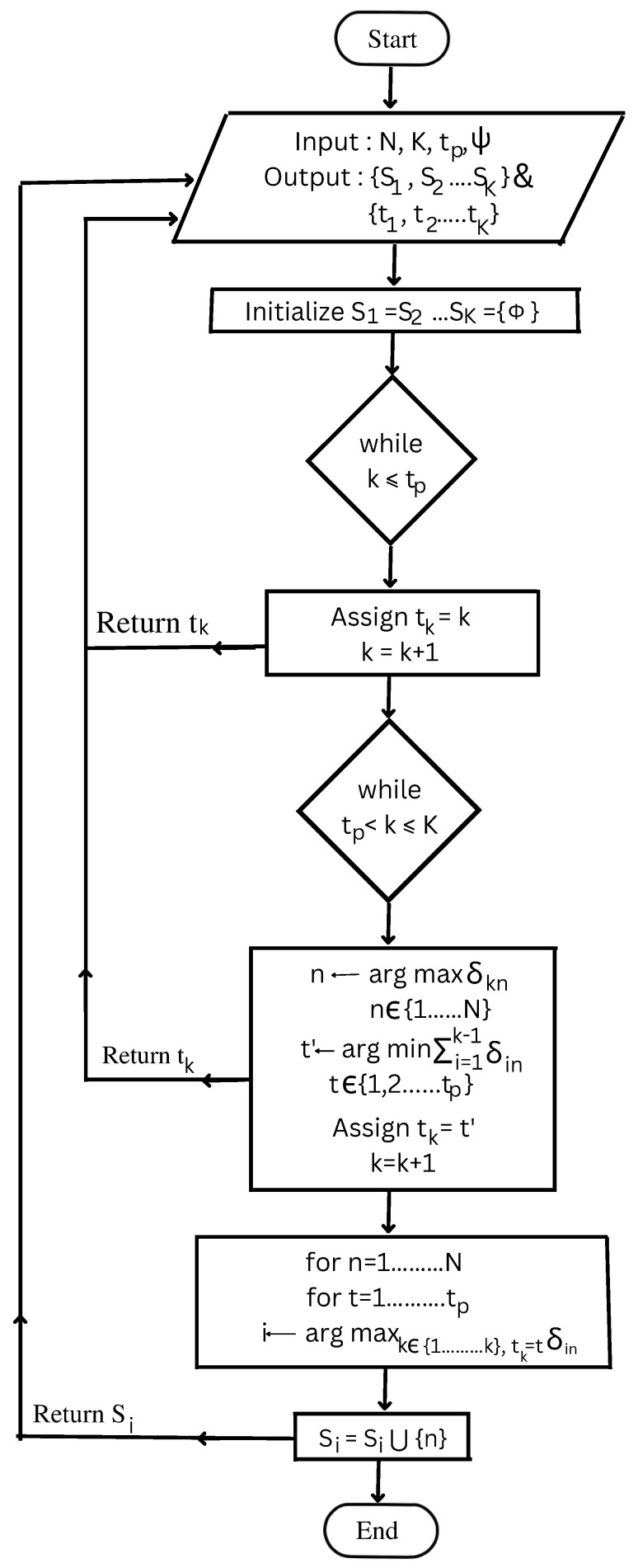
Flowchart of the proposed algorithm.

The pilot-based AP selection (PBAS) algorithm selects subsets of APs $S_{1}$, $S_{2}$, …$S_{K}$ from the pool of *N* APs geographically distributed to serve all *K* user nodes residing in that area. A particular user node connects with those APs, which provides the strongest channel gain for a given pilot. The steps involved in the selection procedure are given in Algorithm 1. The algorithm first allocates the pilots to all the users. The first $t_{p}$ users are assigned the mutually orthogonal $t_{p}$ pilots. For the allocation of pilots to the remaining users, the algorithm first selects the AP *n* with the strongest channel gain to user node *k*. All the user nodes select their master APs using maximum channel gain criteria. After that, out of the $t_{p}$ mutually orthogonal pilots, pilots are assigned to each of the $K-t_{p}$ nodes. For each node–AP pair, the pilot $t^{\prime }$ that node *k* transmits to the AP *n* is determined such that interference at the AP *n* is minimal. The pilot $t^{\prime }$ is assigned to that particular user. This is repeated until the pilot assignment is completed for all *K* users. Next, for all the users assigned the same pilot *t*, the channel gains with AP *n* are evaluated. The user node *i* with the maximum channel gain to AP *n* with pilot *t* is identified. In the end, the AP *n* will be added to subset $S_{i}$ containing the APs serving user node *i*. The process repeats until all the subsets, $S_{1}$, $S_{2}$…$S_{K}$, are evaluated. A flowchart of the proposed algorithm is shown in Figure 2.
**Algorithm 1** PBAS Algorithm**Input**: *N*, $t_{p}$, ψ, *K***Output**: $S_{1}$, $S_{2}$, …$S_{K}$ and $t_{1}$, $t_{2}$ …$t_{K}$1. Initialize $S_{1}=S_{2}=\ldots S_{K}=\left\{\phi \right\}$.2. Allocate the $t_{p}$ orthogonal pilots to the first $t_{p}$ usersAssign $t_{k}\leftarrow k$Repeat step 2 for all $t_{p}$ users3. Allocate pilots to the remaining users ($t_{p}$ + 1 to *K*)4. Select the AP *n* with the strongest channel to the user node *k*$n\leftarrow {arg\;max\;}_{n\in \left\{1,\ldots N\right\}}{\;\;\delta }_{kn}$5. For user *k*, find the pilot $t^{{}^{\prime }}$ that provides minimum interference at the selected AP *n*Assign the user *k* to that pilot $t^{{}^{\prime }}$$t^{\prime }\leftarrow {arg\min}_{t\in \left\{1,\ldots ,t_{p}\right\}}\sum_{i=1,t_{i}=t}^{k-1}\delta_{in}$Assign $t_{k}\leftarrow t^{\prime }$Repeat step 5 to step 7 until *k* = *K* is reached6. The user node that will provides the minimum interference at AP *n* using pilot *t* is identified.$i\leftarrow {arg\;max\;}_{k\in \left\{1,\ldots K\right\},t_{k}=t}{\;\;\delta }_{kn}$7. Add the AP *n* to the subset $S_{i}$ such that$S_{i}=S_{i}\cup \left\{n\right\}$Repeat steps 8–9 for every AP *n* and every pilot *t*8. Return $S_{i}$ and $t_{k}$

## 4. Results and Discussion

This section presents the results of the performance evaluation of the proposed communication model. A geographical network area of 1000 m × 1000 m is considered with the random deployment of APs. The users are uniformly distributed in the given network. The parameters considered for the system model are listed in Table 1. The system is simulated in MATLAB, for which the number of realizations are taken to be $10^{4}$. To compare the proposed system model with other transmission schemes, the simulation setup for scheme 2 considers the coverage area of each AP to be 100 m × 100 m. The network area is divided into small cells, each of 100 m × 100 m dimensions with one AP at the center of the cell. The user nodes in that area are served by the AP located at the center of the small cell [15]. For scheme 1, a single AP with multiple co-located antennas is located at the center, which serves the number of user nodes in that area: mMIMO case [17]. The coverage area of AP is taken to be 500 m × 500 m in the simulation setup of transmission scheme 1.

**Figure 3:**
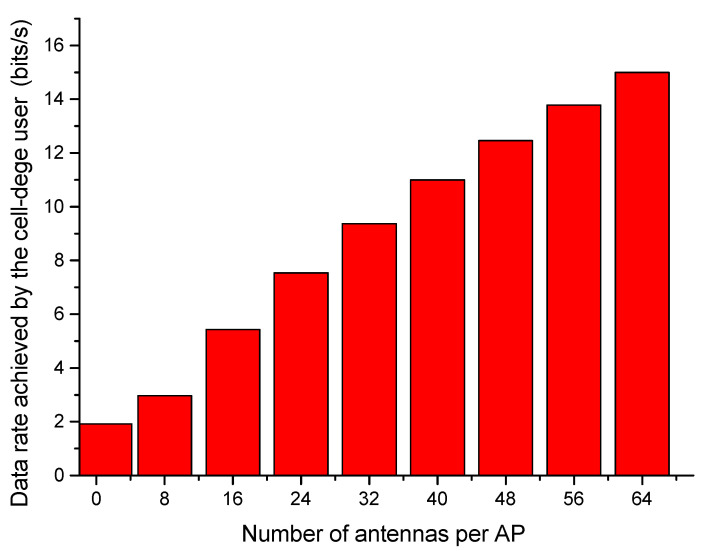
Data rate achieved by the cell-edge user node as a function of number of antennas per AP.

The data rate achieved by a user in the given geographical space is proportional to the number of serving APs in that space. Also, the achieved data rates vary for the cell-center users, intermediate users and cell-edge users. The APs with multiple antennas outperform the APs with a single antenna to provide coverage to all users particularly cell-edge users. The impact of equipping the AP with multiple antennas on the achieved data rate of cell-edge user is depicted in Figure 3. The more antennas there are, the higher the data rate achieved by the cell-edge user. Multi-antenna APs enable better reception in the node’s data stream and improve the data-detection ability of the AP. The data rate achieved by a user node located at the boundary, away from the serving AP, improves with the addition of more antennas to the AP.

The proposed system model considers 100 APs, each equipped with four antennas distributed in a given geographical area to serve the users in that area. MMSE channel estimation is used to find the channel estimates between an AP and a user node. These channel estimates are utilized to find the subset of APs to serve a particular user, as defined in the proposed AP selection algorithm in Section 3. Different receive-combining techniques are used to obtain the performance evaluation.

**Figure 4:**
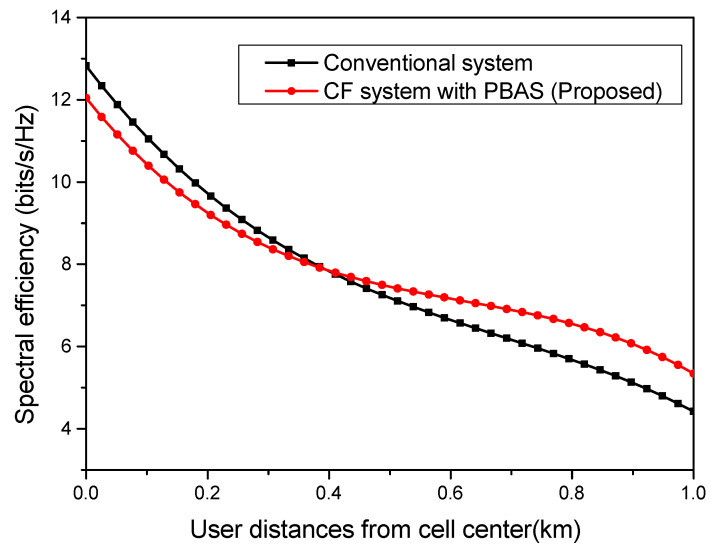
Spectral efficiency as a function of user locations.

Figure 4 illustrates the system performance in terms of the spectral efficiency achieved using the proposed cell-free (CF) model, in which each user node is served through AP cooperation within the clusters. Using the proposed PBAS algorithm, a subset of APs is selected for a given user node. Also, a comparison with the conventional system model is shown, where all APs serve a given user node [30]. MMSE receive-combining is used to carry out the performance comparison. It is observed that the conventional system is better suited to cell-center user nodes, while the proposed system offers an improved performance at the cell edges. Selecting a few APs to serve a particular user instead of using all APs increases the spectral efficiency of the system. For the cell-edge user node, the spectral efficiency improves by 22%, while for the cell-center user node, the value stands at 1.5%.

**Figure 5:**
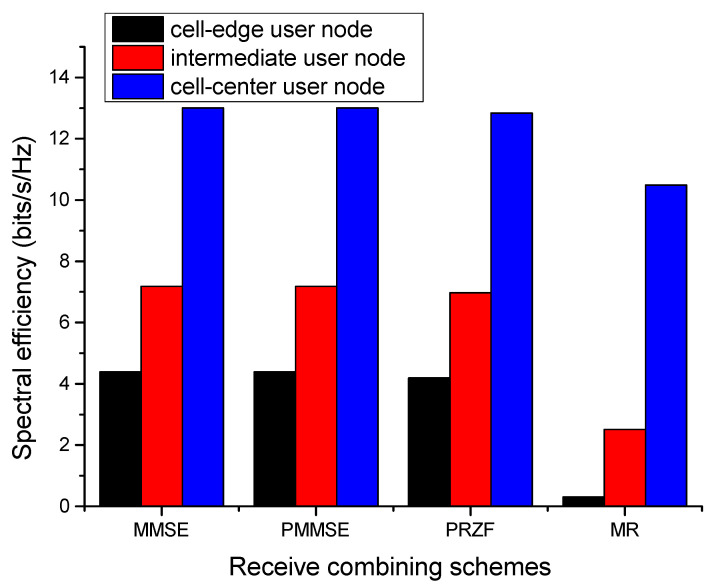
Performance of different receive-combiners with the proposed algorithm.

The performance of different receive-combiners is evaluated in Figure 5 for the proposed communication model using the proposed AP selection algorithm. It has been observed that MMSE receive-combining performs at par with PMMSE-combining, achieving a spectral efficiency of 4.3943 bits/s/Hz for the cell-edge user node and 13.0088 bits/s/Hz for the cell-center user node. The lowest performance is obtained with MR-combining, which provides a minimum spectral efficiency of 0.3077 bits/s/Hz and maximum of 10.4858 bits/s/Hz. The PRZF combining outperforms MR-combining and offers an improvement of 22.4% in the spectral efficiency achieved by cell-center users.

**Figure 6:**
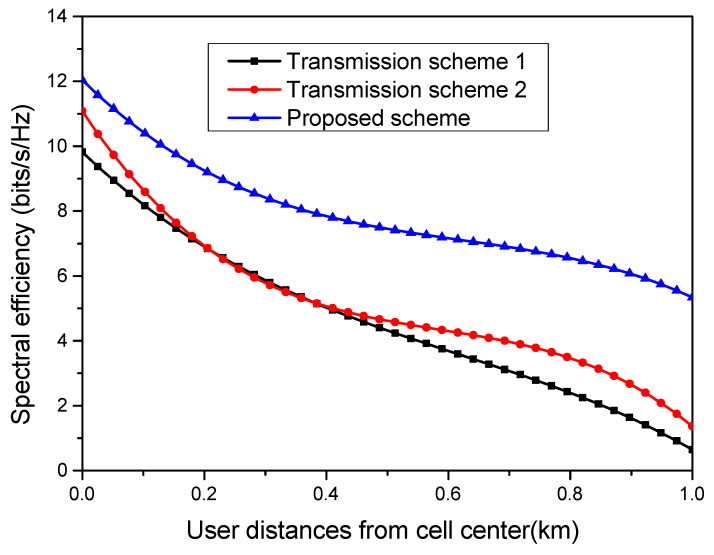
Comparison of different schemes for spectral efficiency.

Three communication scenarios are compared in Figure 6 in terms of their spectral efficiency performance. In the first transmission model, one multi-antenna AP serves all the users distributed in a given geographical area [17]. In the second simulation model, the area is divided into small cells with one AP per cell, which is considered as small-cell transmission model [15]. In the proposed system model, a subset of APs cooperate among themselves in order to serve a particular user. The proposed algorithm creates clusters of APs, with each cluster serving a particular user. It is clear from Figure 6 that the proposed transmission model achieves the highest spectral efficiency and outperforms the other two transmission models. Transmission scheme 2, which is a small-cell model, is more suited to user nodes adjacent to the APs and nodes at the boundaries. For intermediate user nodes, transmission schemes 1 and 2 have a similar performance in terms of the achieved spectral efficiency. Also, the spectral efficiency attained by a user node located at the edge in the proposed transmission model is 5.3676 bits/s/Hz, compared to the 0.756 bits/s/Hz and 1.0501 bits/s/Hz of spectral efficiency attained by the edge node in the first and second transmission models, respectively.

**Figure 7:**
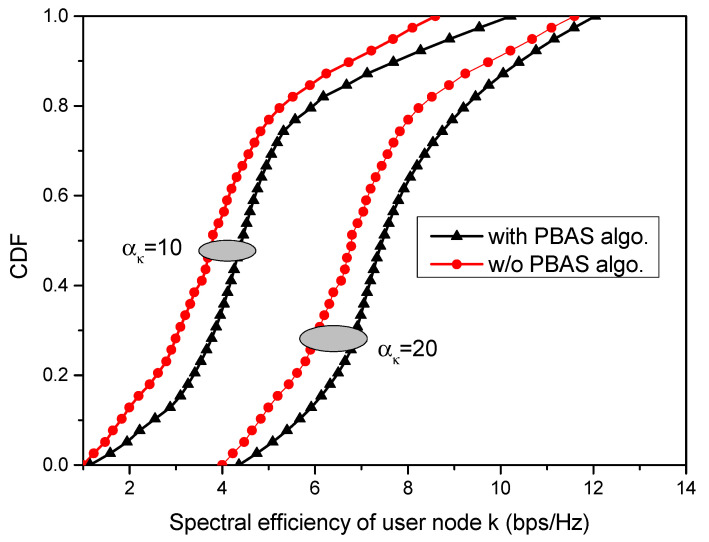
CDF of the spectral efficiency achieved by node k with and without the PBAS algorithm.

To assess the performance of the proposed AP selection algorithm, the cumulative distribution function (CDF) of the spectral efficiency achieved by node *k* is obtained for different transmit powers and plotted in Figure 7. It is observed that the user node *k* achieves better spectral efficiency with the incorporation of the proposed PBAS algorithm compared to its performance in a system with no AP selection. There is an improvement of 9.27% in the spectral efficiency achieved by the cell-edge user node with the AP selection algorithm, with $\alpha_{k}$ = 20 dBm over $\alpha_{k}$ = 10 dBm. However, with small transmit powers, the gain in spectral efficiency achieved with AP selection is greater for cell-center nodes.

## 5. Conclusions

The massive number of connected devices and the huge data flow require intelligent networks capable of handling extreme data traffic and delay-sensitive communication with targeted computational accuracy. This paper proposes a dynamic cell-free framework for a 6G-enabled IoT network, which offers massive connectivity and network coverage. In this transmission model, a large number of APs are distributed in a given area to serve a large number of user nodes. The simulation setup is modeled in MATLAB to evaluate the system performance in terms of the achieved data rate and spectral efficiency. For the proposed network scenario, an AP selection algorithm, PBAS, is proposed, which selects a subset of APs for a particular node based on pilots. Using a few APs per node against all APs results in improved spectral efficiency. The spectral efficiency achieved by an edge node improves by 22%, while the cell-center user node shows an improvement of 1.5%. The effect of using different receive-combiners on the system performance is also shown for different user locations. MMSE receive-combining performs at par with PMMSE-combining, offering a maximum spectral efficiency of 13.0088 bits/s/Hz, while MR-combining achieves the lowest performance, offering a maximum of 10.4858 bits/s/Hz. Further, the proposed simulation model is compared with two other simulation setups. The first setup uses one AP for all users while the second setup uses one AP for one user. The analysis shows that the proposed transmission model, allowing only a set of APs to communicate with a particular user, achieves the highest spectral efficiency and outperforms the other two transmission models. The spectral efficiency attained by a user node located at the edge of the proposed transmission model is 5.3676 bits/s/Hz compared to the 0.756 bits/s/Hz and 1.0501 bits/s/Hz of spectral efficiency attained by the edge node in the first and second transmission models, respectively. The variation in the achieved spectral efficiency for different transmit powers suggests that the gain in spectral efficiency achieved with AP selection is greater for cell-edge nodes with high transmit powers.

## Figures and Tables

**Table 1:** Parameters considered for performance evaluation.

Parameters	Value	Parameters	Value
*N*	100	$t_{p}$	10
*M*	4	$H^{\prime }$	10 m
*K*	40	$\alpha_{k}$	100 mW
*B*	20 MHz	$\sigma^{2}$	−94 dBm
$t_{c}$	2 ms	$B_{c}$	100 kHz
$\tau_{c}$	200		

## Data Availability

Not applicable.

## Abbreviations

The following abbreviations are used in this manuscript:

5G	Sixth Generation
6G	Sixth Generation
IoT	Internet of Things
AP	Access Points
BS	Base Station
MIMO	Multiple-Input–Multiple-Output
IoE	Internet of Everything
RF	Radio Frequency
IoV	Internet of Vehicles
TDD	Time Division Duplex
CPU	Central Processing Unit
IRS	Intelligent Reflecting Surfaces
IIoT	Industrial IoT
SWIPT	Simultaneous Wireless Information and Power Transfer
IoUT	Internet of Underwater Things
SE	Spectral Efficiency
EE	Energy Efficiency
SNR	Signal-to-Noise Ratio
SINR	Signal-to-Noise-Plus-Interference ratio
CSI	Channel State Information
LSFD	Large-Scale Fading Decoding
MR	Maximal Ratio
PMMSE	Partial Minimum Mean Square Error
PRZF	Partial Regularized Zero Forcing
